# Non-invasive imaging techniques for predicting healing status of diabetic foot ulcers: a ten-year systematic review

**DOI:** 10.3389/fmedt.2025.1648973

**Published:** 2025-09-29

**Authors:** Nila N. Sari, Quoc C. Ngo, Nemuel D. Pah, Rajna Ogrin, Elif Ekinci, Akram Hourani, Barbara Polus, Dinesh K. Kumar

**Affiliations:** ^1^ School of Engineering, RMIT University, Melbourne, VIC, Australia; ^2^ Electrical Engineering, Politeknik Negeri Bandung, Bandung, Indonesia; ^3^ Electrical Engineering, Universitas Surabaya, Surabaya, Indonesia; ^4^ Bolton Clarke Research Institute, Melbourne, VIC, Australia; ^5^ Department of Medicine, University of Melbourne, Parkville, VIC, Australia; ^6^ Australian Centre for Accelerating Diabetes Innovations (ACADI), Department of Medicine, University of Melbourne, Melbourne, VIC, Australia; ^7^ Department of Endocrinology, Austin Health, Heidelberg, VIC, Australia

**Keywords:** diabetes-related foot ulcer (DFU), imaging techniques, prediction, healing, systematic review

## Abstract

**Introduction:**

Early and accurate detection of diabetes-related foot ulcers (DFU) that may become chronic is essential to prevent long-term disability, amputation, and mortality. Various non-invasive imaging techniques have been developed to detect and monitor DFU progression, but none have yet been widely adopted in clinical practice. This review summarizes current advancements in non-invasive image techniques for DFU wound healing prediction and identifies research directions to support clinical translation.

**Methods:**

A systematic, multi-disciplinary review was conducted focusing on three imaging methods: photographic, hyperspectral, and thermal imaging. Articles published between July 2014 and July 2024 were searched across five databases: PubMed, Scopus, CINAHL, Embase, and Web of Science. The search was limited to English-language, peer-reviewed journal articles. The review followed PRISMA guidelines and applied the CASP quality appraisal tool.

**Results:**

The initial search identified 2,937 articles, of which 22 studies met the inclusion criteria, including 17 original studies (9 medical and 8 engineering) on DFU healing prediction using imaging techniques and 5 relevant review articles.

**Discussion:**

Each imaging method offers specific benefits and faces unique limitations: photographic imaging is user-friendly but lighting-sensitive; thermal imaging reflects inflammation but requires multimodal integration; hyperspectral imaging provides biochemical insight but is costly and less portable. Visual and thermal imaging, in particular, demonstrate strong potential for early and real-time prediction when combined with machine learning/deep learning. These methods offer portability, ease of use, and potential for automated analysis on a single device, making them suitable for clinical and community settings. However, challenges such as standardization and integration complexity remain. Continued research with larger datasets and improved validation is needed to enhance clinical readiness.

## 1 Introduction

Diabetes mellitus is a chronic metabolic disorder characterized by elevated blood glucose levels, which can lead to several complications if not optimally managed. Among these, diabetes-related neuropathic foot ulcers pose a substantial public health concern (1–3). Recent reports show that the lifetime risk of developing a diabetes-related foot ulcer (DFU) among individuals with diabetes ranges from 19% to 34% (4). This number highlights the importance of preventive care and regular monitoring of DFUs as an integrative treatment in diabetes care. DFUs are a leading cause of non-traumatic lower limb amputation and significantly increase the risk of infection, reduce quality of life, and impose a substantial economic burden on both healthcare systems and people living with diabetes (5, 6).

The most common cause of foot ulcers in people living with diabetes is trauma in an insensate foot due to peripheral neuropathy (7). Currently, an estimated 20% of DFUs do not heal in the expected trajectory, increasing the risk of complications such as limb amputations (4). The current best clinical practice to identify whether a wound will become chronic, i.e., a wound that will not heal within 12 weeks, is based on the area of the wound being reduced by half in the first four weeks. However, there are several issues with this method. It requires waiting 4 weeks to ascertain wound healing ability, thereby delaying intervention and increasing the risk of complications developing. Further, wound area measurement is generally undertaken by tracing the wound using acetate, necessitating physical contact with the wound and increasing the risk of infection. In addition, a recent review of the literature has suggested that this approach has little evidence to support it (8).

Conventional imaging methods like Magnetic Resonance Imaging (MRI), Computed Tomography (CT), and Positron Emission Tomography (PET) are still used to check for infection or bone damage in DFU, but they are impractical for routine outpatient use or for predicting DFU healing trajectories (9). The limitations of these images include radiation exposure (in CT and PET), long scan times, and limited availability (10–13). Recent advancements in non-invasive imaging technologies have increased options to predict wound healing, with researchers reporting on various imaging techniques for assessing and monitoring DFUs. These include visual [Red Green Blue (RGB)] imaging, thermal imaging, and hyperspectral imaging modalities. While the focus of these technologies may vary, the main aim is to enable clinicians to accurately assess DFUs, including predicting the ability to heal in the normal trajectory. The results reported show that these imaging techniques, when used with analysis algorithms and software, were more accurate when compared to traditional paper-based documentation of wound assessment methods, which are based on measuring change in the area of the wound, and thus have the potential to enhance clinical care (14). While the research outcomes are very positive, none have yet been integrated into standard clinical practice. It is also not evident which of the imaging modalities are most suitable to be used in clinical, community, and home care services, and what the limitations are reported in the literature.

In clinical studies, advancements in nanoparticles and drug delivery systems (15–17) have been developed in chronic wound care. Raghunanth et al. (18), demonstrated a promising oral delivery system for insulin using chitosan-coated solid lipid nanoparticles (Ch-Ln-SLNs) with piperine, which significantly improved glycemic control and could potentially influence DFU healing. Sarma et al. (19) reviewed the use of electrospun nanofibers for wound dressing that supports controlled drug release and tissue regeneration for chronic wound therapy. Ahikiriza et al. (20) highlighted the therapeutic potential of Ugandan natural products for managing diabetes. Additionally, imaging techniques, such as thermal and hyperspectral imaging can non-invasively monitor the local wound response to such therapies by detecting changes in perfusion, oxygenation, and inflammation. The integration of advanced nanoparticles and imaging technologies like thermal and hyperspectral imaging could enhance diagnostic accuracy and therapeutic monitoring in chronic wounds, including diabetic foot ulcers.

Existing studies primarily focus on broad imaging techniques but lack a targeted exploration of the potential to predict DFU wound healing outcomes in those technologies where this could be an effective application. A systematic review and meta-analysis of tools for predicting wound healing in DFUs by Wang et al. (21) reported that most studies up to October 2011 relied on transcutaneous oxygen measurement (TcPO2) and ankle-brachial index (ABI) as prognostic tools from hyperspectral imaging analysis. In 2015, Paul et al. (22) reviewed optical imaging technologies for wound assessment and highlighted the potential areas for future exploration. More recently, Chan et al. (14) briefly reviewed progress in wound assessment, imaging, and monitoring systems but did not specifically address DFU healing prediction. Another recent review by Saiko et al. (23) investigated 32 years of research on the use of Hyperspectral Imaging (HSI) in wound care, including DFU applications, but did not compare different imaging modalities for predictive effectiveness. Similarly, a recent narrative review by Godavarty et al. (9) provided an extensive overview of conventional and optical imaging techniques for DFU, including a novel spatiotemporal near-infrared (NIR) based approach. However, the review was not systematic and did not identify the most suitable imaging modality for DFU wound healing prediction. Overall, none of these reviews offer a comparative framework to identify a suitable path that will result in the adoption of imaging technologies to predict wound healing of DFU. Table 1 presents a comparative summary of our systematic review against previous literature.

**Table 1:** Comparison of this review with previous reviews.

Reference	Year	Focus Area	Healing progression/prediction Analysis Techniques	Non-invasive imaging modalities	Features	Machine Learning/Deep Learning	Limitation	Future Direction
Paul *et al*. (22)	2015	Optical imaging for wound assessment	☑	Laser doppler imaging, thermal imaging, near-infrared spectroscopy, spectral imaging, ultrasonography	☑	⮽	≈	≈
Wang et al. (21)	2016	Screening test to predict wound healing	☑	Hyperspectral imaging	☑	⮽	☑	≈
Chan et al. (23)	2020	Imaging technique wound assessment	☑	Hyperspectral imaging, spectroscopy imaging, fluorescence imaging	☑	≈	☑	☑
Saiko et al. (14)	2020	Hyperspectral imaging in wound care	☑	Hyperspectral imaging	☑	⮽	☑	☑
Godavarty (9)	2023	Future directions of DFU imaging	☑	Hyperspectral imaging (HIS), Multispectral imaging (MSI), Near-infrared spectroscopy (NIRS), Diffuse reflectance spectroscopy (DRS), Laser Doppler flowmetry/imaging (LDF/LDI)	☑	⮽	☑	☑
**This paper**	**2025**	**Non-invasive Image Techniques Wound Healing Prediction**	☑	**Photograph imaging, Thermal imaging, Hyperspectral imaging**	☑	☑	☑	☑

☑, discussed; ≈, partially discussed; ⮽, not discussed.

This systematic, multi-disciplinary review aims to identify what is the most feasible imaging technique that can be used for predicting the healing status of DFU in clinical, community, and home care settings with a focus on three primary imaging methods: visual imaging (RGB), hyperspectral imaging, and thermal imaging. The review will address the following research questions:

• What are the strengths and weaknesses of the imaging techniques for predicting the healing status of diabetes-related foot ulcers?
• How do different wound characteristics captured by non-invasive imaging compare for developing healing prediction models?
• Is research in this field mature for the into clinical practice?

## 2 Methodology

**Figure 1:**
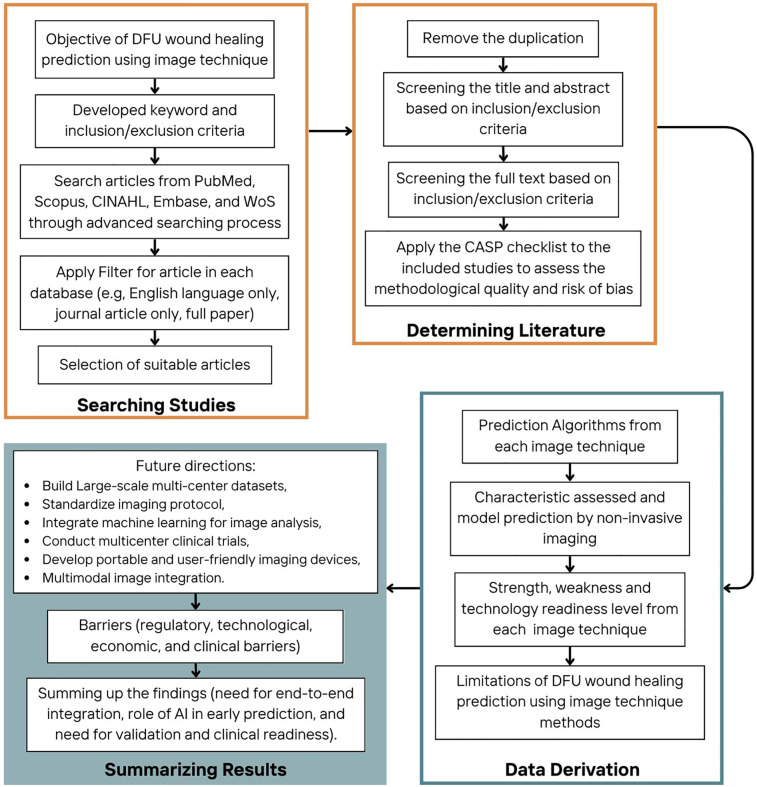
Flow chart of systematic review.

The process for evaluating DFU wound healing prediction using non-invasive image techniques followed a structured approach, divided into four main stages: searching strategy, determining literature, data derivation, and summarizing results, as can be seen in Figure 1. A list of acronyms used in this paper is given in Table 2.

**Table 2:** List of acronyms.

Acronyms	Definition
ABI	ankle-brachial index
ANN	Artificial Neural Network
ANOVA	Analysis of Variance
AUROC	Area Under the Receiver Operating Characteristic
CT	Computer Tomography
DeoxyHb	Deoxyhemoglobin
DFU	Diabetes Related Foot Ulcers
GA	Granulation area
GI	Granulation Index
HBOT	Hyperbaric Oxygen Therapy
HIS	Hyperspectral Imaging
HUI	Healing Ulcer Index
MRI	Magnetic Resonance Imaging
NIR	Near-Infrared Perfusion
O2Sat	Oxygen Saturation
OxyHb	Oxyhemoglobin
PCA	Principle component analysis
PET	Positron Emission Tomography
RF	Random Forest
RGB	Red, Green, Blue
R-CNN	Region-Based Convolutional Neural Network
ROC	Receiver Operating Characteristic
ROI	Region of Interest
RPN	Region Proposal Network
RYB	Red Yellow Blue
SNN	Siamese Neural Network
SpO2	Oxygen saturation
SPSS	Statistical Package for the Social Sciences
StO2	Tissue oxygen saturation
SVM	Super Vactor Machine
TBI	Toe-brachial index
TcPo2	Transcutaneous Oxygen Measurement
THI	Tissue Hemoglobin Index
TWI	Tissue Water Index
WA	Wound Area
WMA	Wound Margin Advance

### 2.1 Searching strategy

In this stage, the primary objective was to identify relevant studies on DFU wound healing prediction using imaging techniques. PRISMA guidelines were used to identify journal articles published in the last decade, (July 2014 to July 2024) that reported wound healing prediction algorithms utilizing imaging techniques. Publications were obtained from five online databases that included engineering and clinical journals (Web of Science, Scopus, CINAHL, Embase, and PubMed) using an advanced search process. The following search terms, developed in consultation with the university librarian, were used; (“diabetic foot” OR “diabetic foot ulcers” OR “diabetic-related foot ulcers” OR “neuropathic diabetic foot ulcer”) AND (“imaging technique” OR “imaging system” OR “hyperspectral imaging” OR “thermal imaging” OR “photographic imaging”) AND (“prediction” OR “predicting” OR “predict”) AND (“healing status” OR “healing time” OR “healing”). Only English-language journal articles were included; non-English studies were excluded due to resource limitations for accurate translation. Preprints and unpublished data were not considered, as filters were applied during the initial database search to include only peer-reviewed, published journal articles. This approach ensured that all included studies went through formal scientific review, reduced the risk of bias associated with unverified or incomplete findings.

### 2.2 Determining literature

**Figure 2:**
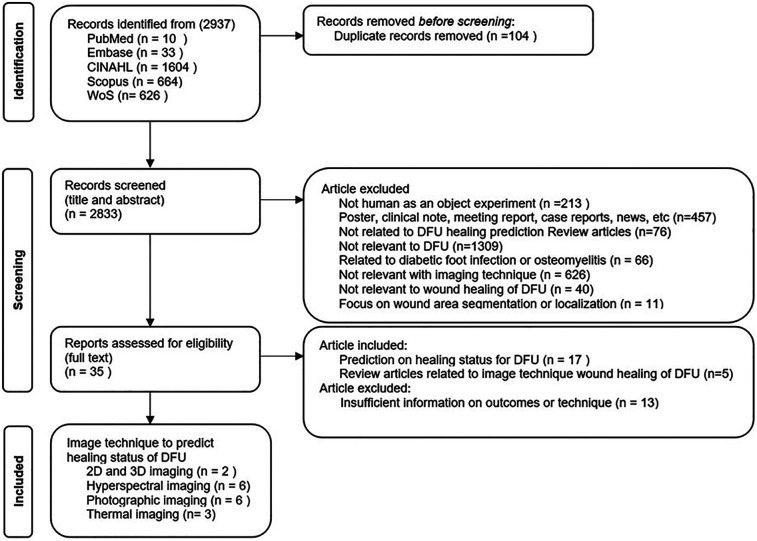
List of inclusion and exclusion criteria using PRISMA guideline.

After collecting the studies, the next stage involved screening the articles. A PRISMA flowchart is shown in Figure 2, which was used to identify the articles. EndNote (Clarivate, UK) was used to identify and remove duplicates. After the removal of 104 duplicates, the searching process identified 2,833 publications to be considered for the screening step.

The screening process was conducted in two steps. In the first step, the title and abstract of each study were reviewed by two reviewers (NNS, NDP) to generate a list of relevant studies based on the inclusion and exclusion criteria. Grey literature, including posters, clinical notes, meeting reports, case reports, and news articles (*n* = 457), was excluded to maintain consistency in quality assessment and ensure inclusion of studies that went through the peer review process. The exclusion criteria included; studies that did not involve human subjects (*n* = 213); studies that not related to DFU healing prediction review articles (*n* = 76); studies not relevant to DFU (*n* = 1,309); those focused on diabetic foot infections or osteomyelitis (*n* = 66); studies that did not utilize imaging techniques (*n* = 626); those unrelated to DFU wound healing or healing prediction (*n* = 40); that focused on wound area segmentation or localization only (*n* = 11).

If the information from the title and abstract is insufficient to decide on the inclusion or exclusion of a study, the full text was reviewed. The second step involved a full-text review by the two reviewers (NNS, NDP) to obtain the final list of studies for data extraction. The inclusion criteria were: (1) original articles focused on predicting the healing status of diabetic foot ulcers (DFU) using imaging techniques (*n* = 17) and (2) review articles discussing imaging methods relevant to DFU wound healing (*n* = 5). Studies with insufficient information on outcomes or imaging methodology were excluded (*n* = 13). Any conflicts between the two reviewers were resolved by input from a third reviewer (QN).

After the two-stage screening process, as shown in Figure 2, 22 publications (17 journal articles and 5 systematic reviews) were identified. Nine articles were from clinical journals, eight were from engineering journals, and five were review articles, all of which were from the clinical domain. The CASP (Critical Appraisal Skills Programme) checklist for appraising quantitative studies was used for a quality checklist of included articles as shown in Table 3. Fifteen articles met the criteria for high-quality studies, while two met fewer criteria. No studies were removed based on quality because all were regarded as giving valuable insight.

**Table 3:** CASP checklist for appraising quantitative studies.

No	CASP question	(25)	(26)	(27)	(28)	(29)	(30)	(31)	(32)	(33)	(34)	(35)	(36)	(37)	(38)	(39)	(40)	(41)
1	Was the research question clearly defined?	Y	Y	P	Y	Y	P	Y	Y	Y	Y	Y	Y	Y	Y	Y	Y	Y
2	Was the study design appropriate?	Y	Y	P	Y	Y	Y	Y	Y	Y	Y	Y	P	Y	Y	Y	Y	P
3	Was the sample size justified?	Y	Y	N	N	Y	Y	N	Y	Y	Y	Y	N	Y	Y	Y	Y	Y
4	Were the inclusion/exclusion criteria clear?	Y	Y	Y	Y	Y	Y	Y	P	Y	Y	Y	N	Y	Y	Y	Y	P
5	Were the methods of measurement valid and reliable?	Y	Y	P	Y	Y	Y	P	Y	Y	Y	Y	P	Y	Y	Y	Y	Y
6	Was the statistical analysis appropriate?	Y	Y	P	P	Y	Y	Y	Y	Y	Y	Y	N	Y	Y	Y	Y	Y
7	Were the outcomes clearly defined?	Y	Y	P	Y	Y	Y	Y	Y	Y	Y	Y	Y	Y	P	Y	Y	Y
8	Was the study ethical?	Y	Y	Y	Y	Y	Y	Y	Y	Y	Y	Y	P	Y	Y	Y	Y	Y
9	Were the findings applicable to other settings?	N	P	P	N	Y	P	P	Y	P	P	P	P	P	P	P	P	P
10	Are the results reliable?	Y	Y	P	Y	Y	P	P	Y	Y	Y	Y	P	Y	Y	P	Y	Y

Y, yes; P, partial; N, no.

While only 17 studies were included out of 2,937 screened, this targeted selection allowed for a more in-depth evaluation of advanced image modalities. This focused inclusion ensured that each selected study contributed valuable insights into the prediction of DFU wound healing using non-invasive imaging techniques. This approach aligns with the PRISMA guidelines, which emphasize the importance of methodological rigor in systematic reviews, thus enhancing the reliability and depth of our findings.

From the review, we identified four main categories of wound healing prediction using non-invasive imaging techniques: (a) 3D imaging (25, 26), (b) visual (RGB) imaging (27–32), (c) hyperspectral imaging (33–38), and (d) thermal imaging (39–41). While 3D imaging was not included in the search keywords, it appeared in the results and hence has been included in this study due to the visibility of this device for use in clinical, community, and home care services.

### 2.3 Data Derivation

In this stage, valuable data were extracted from the selected articles. The prediction algorithms used in each non-invasive imaging technique were identified in Section 3, the characteristics for wound healing were assessed in Section 4, and the prediction models were described in Section 5. Additionally, the strengths, weaknesses, and technological readiness levels of each imaging technique were evaluated in Section 6.

### 2.4 Summarizing Results

The final stage involved summarizing the findings from the data derivation process. Key insights were drawn, including future directions for research, open research issues, and the barriers that were faced in clinical practice (Sections 7 and 8). This stage provided a comprehensive summary of findings and future investigations of DFU wound healing management, especially for wound healing prediction using non-invasive imaging techniques.

## 3 Prediction algorithms

Wound healing prediction algorithm based on image technique can be considered according to each different image modality.

### 3.1 Healing prediction using 3D imaging

Measuring the volume of the wound, which includes the depth of the wound has been proposed for better evaluation of the wounds. Two studies reported the use of 3D imaging techniques in DFUs (25, 26). This prediction technique typically follows a series of steps to evaluate wound healing, beginning with capturing the wound image using a specialized 3D camera system. One study involved an adhesive optical target for calibration (26).

Multiple images were captured from multiple angles after wound debridement, and these images were then combined into a single 3D model by the software. The clinicians then manually traced the wound area on the monitor for precise measurements to calculate various parameters such as wound area, volume, and depth. The wound measurements were compared from baseline to predict wound healing progression.

These measurements were analyzed using statistical models such as linear regression, which correlates the changes in wound dimensions with time to healing. Prognostic indicators, such as the healing slope and regression analysis, determined whether the wound was healing as expected or stagnating. One study (25) used measurement from 2D imaging as a comparison, while another (26) used the complete closure of the wound (100% skin regrowth) as a reference.

### 3.2 Healing prediction using visible light imaging

Visible light photographic imaging, generally performed using RGB (Red, Green, Blue) imaging, is one of the imaging techniques that closely resembles traditional assessment in clinical practice due to its focus on measuring physical appearance such as length, width, depth, edge, and peri-wound skin to monitor the wound healing progression (42, 43).

In the list of included articles, six studies focused on using photographic imaging techniques to predict wound healing progression in DFU patients (27–32). The algorithms for predicting healing in DFUs mostly involve four main processes: image acquisition, preprocessing, segmentation, and classification. The algorithm steps used for predicting the healing progression are described below.

#### 3.2.1 Image acquisition

The first step requires capturing the visual light images of the DFU with a controlled image acquisition protocol (31); for example, the wounds were taken at baseline and on days 3, 7, and 14 (29), with the distance between the camera and the wound, camera properties, and the ambient light conditions well described. While the use of smartphone cameras (27, 28) provides ease of data capture, one study proposed a specialized image capture box designed to ensure the consistency of lighting and angles in pictures captured at different time (27).

#### 3.2.2 Pre-processing

After capturing the image, it is necessary to pre-process the images to improve the quality, handle missing pixels or reduce the size, as required for the application. In the reviewed papers, images were pre-processed by decompressing into 24-bit bitmap files, down-sampled to reduce resolution and processing time, and then applied Gaussian smoothing to remove noise (27, 31). In a multi-step approach study (30) that combined clinical data with image processing techniques, the k-nearest neighbor algorithm was used to handle missing data collection, while non-numeric features were discretized using methods such as one-hot encoding (30).

#### 3.2.3 Segmentation

Image segmentation is the process of separating the image into separate segments, often to identify objects or regions of interest. In the segmentation process reported by Wang et al. (27), a mean-shift segmentation algorithm was used to divide the image into homogeneous regions, followed by region fusion to address over-segmentation. The foot outline was detected using skin color thresholds in the CIELAB color space, the guidelines for color representation based on human perception. The wound boundary was identified through connected component analysis (27). In another study, the wound area (WA), granulation area (GA) based color, and the calculation of the granulation index (GI) as the percentage of GA relative to the total wound area were traced and measured using ImageJ software (29). Clinicians also conducted manual segmentation to collect features using image editing software (30). For skin segmentation using the YCbCr color space, morphological operators were applied to refine the image, and a 25 cm^2^ reference square was used for pixel-to-real-area calibration (31).

#### 3.2.4 Classification of wound

The classification of wound area has been used to differentiate between color, size, and tissue type. Five approaches were reported for the process. K-means clustering algorithms were used to divide the segmented wound area into red, yellow, and black tissue, representing healthy, slough/infected, and necrotic tissues, respectively (27). Statistical analysis with SPSS was used to assess the significance of wound size changes and compare the efficacy of different treatments (29). Third, a Random Forest and Support Vector Machine was trained for predictive analytics (30). Fourth, using RGB pixel values and a feedforward multilayer perceptron (or artificial neural network), the ulcer tissues were classified as granulated, dilacerated/fibrin, or necrotic (31). Fifth, a Siamese Neural Network (SNN) was used to assess DFU progression over time by computing distances from each class anchor point (none, infection, ischemia, both, and healthy) and generated a table with figures and a radar chart (32).

### 3.3 Healing prediction using hyperspectral imaging

Hyperspectral imaging (HSI) is a technique that determines the high-resolution spectrum of each pixel across the electromagnetic spectrum of interest. Six studies used HSI to predict the progression of DFU healing (33–38). As the first step, the eligible patients were properly screened based on inclusion criteria (35, 37, 38), for example, by abstaining from smoking or caffeine (33). To reduce noise and normalize attenuation values, the calibration of a push-broom camera across a wavelength range of 430–750 nm was applied in the study conducted by Yang et al. (34). Hyperspectral imaging data, corrected for light scattering (33), was processed to calculate tissue oxygenation levels (33), with skin temperature and the ankle-brachial pressure index (ABPI) recorded as baseline measurements (33). In another study, baseline assessments included transcutaneous oxygen pressure (TcpO2), ankle-brachial and toe-brachial indexes, and HSI, which evaluated tissue parameters such as oxygen saturation, hemoglobin index, near-infrared perfusion, and water content (37). The HSI was also used to quantify peri-wound oxyhemoglobin (OxyHb), deoxyhemoglobin (DeoxyHb), and oxygen saturation (O2Sat) in two studies (36, 38). Photonics-based algorithms were used in one study (36) and included the combination of HSI and thermal imaging applied to capture data on biomarkers including OxyHb, DeoxyHb as well as foot temperature patterns to compute the oxygenation metrics Oxygen saturation (SpO2) and Tissue oxygen saturation (StO2).

Monitoring of wound healing progression was carried out at 12 weeks (33, 38) and 24 weeks (33, 37), while other studies conducted monitoring over a 3-week period to assess changes in wound size, perfusion [e.g., transcutaneous oxygen levels (36), hyperspectral imaging], and cytokine levels (35). Using these features, wounds were classified as “healers” or “non-healers,” and a predictive model was created to estimate the likelihood of wound healing. In another study (37) healing was defined as complete epithelization without drainage sustained for at least 10 days within 24 weeks.

### 3.4 Healing prediction using thermal imaging

Thermal imaging records the infrared or near-infrared radiation of the object to assess its thermal condition. It is a non-invasive technique that detects temperature variations on the surface of an object. In the prediction of wound healing of DFU, in the study by (28) thermal images were segmented into isothermal patches to analyze the wound boundaries and the areas according to the temperature distribution (39). The assessment parameters include the temperature of the wound bed, the area of the isothermal patch on the wound bed, the area of the isothermal patch surrounding the wound and the number of isolated isothermal patches within the wound region. The physical area of the wound bed was determined from color images (39).

In another study (40), the combination of thermal imaging and computerized planimetry was used to first monitor the temperature change indicative of metabolic activity and inflammation, and second to measure wound surface area and wound perimeter. This study integrated these measurements to evaluate the effect of Hyperbaric Oxygen Therapy (HBOT) (40).

Deep learning-based methodology was used in the combination of thermal and visible imaging (41). Experts annotated the fused image as an input to the Mask R-CNN model for precise wound segmentation. Weekly images were analyzed to calculate metrics (39–41) including ulcer-to-foot area ratio and mean temperature distribution. These metrics were correlated with clinician-provided ground truth data to track the healing progression.

## 4 Wound characteristics assessed by non-invasive imaging

The literature describes three methods for assessing the healing condition of wounds: physiological, geometric, and surface assessment.

### 4.1 Physiological assessment

Evaluating the physiological aspects of wounds by hyperspectral imaging is essential for forecasting healing trajectory. This assessment concentrates on internal tissue properties and physiological states. Wound healing is a complex and dynamic process that involves four connected stages: blood clotting (hemostasis), inflammatory response, tissue growth (proliferation), and tissue repair (44–46). A proper understanding of the four stages of wound healing is essential for the physiological assessment of wounds (47, 48).

#### 4.1.1 Tissue oxygenation

This parameter is one of the critical predictors of healing status for DFUs in HSI due to its fundamental role in wound repair processes (33–38). Adequate oxygen, a key component for cellular respiration, is important for various stages of wound healing (49, 50). Higher tissue oxygenation levels correlate with better wound healing outcomes in diabetic patients, whereas hypoxia impairs healing (49). Measurements of wound oxygenation, such as transcutaneous oxygen measurements (TcpO2), can guide treatment planning and predict healing potential (50).

#### 4.1.2 Tissue hemoglobin

The tissue hemoglobin index serves as a predictor of healing status in DFUs in HSI due to its ability to assess microvascular oxygenation and perfusion, which are crucial factors in wound healing (37). The cutaneous tissue hemoglobin oxygenation can be quantified from hyperspectral imaging technology. The technology generates anatomically relevant tissue oxygenation maps that correlate with wound healing potential (49). Studies have shown that higher oxyhemoglobin levels and oxygen saturation in the peri-wound area are associated with better healing outcomes (49, 51).

#### 4.1.3 Temperature

Temperature monitoring of DFUs is a predictor of healing status due to its ability to reflect local blood supply, tissue damage, and inflammatory processes (39). Higher wound temperatures typically indicate infection or inflammation, while decreased temperatures suggest impaired healing (52, 53).

### 4.2 Geometrical assessment

Geometric wound assessment is an important predictor of healing status for DFUs, including the shape, wound size, perimeter, surface area, and volume measurements (25, 26, 43, 54, 55). These quantitative metrics provide objective and reproducible data that can help clinicians monitor wound progression over time, evaluate treatment efficacy, and predict healing outcomes (56, 57). The linear nature of wound margin advance (WMA) over time, calculated as the change in wound area divided by wound perimeter, has been shown to be an effective predictor of healing in DFUs (56). Medical professionals can use this linear pattern to estimate the total healing time based on assessments undertaken at 4–5 weekly intervals. This capability makes it a valuable asset for timely intervention and fine-tuning treatment approaches (56). Studies have shown that a 40% reduction in wound surface area within the first two to three weeks of treatment predicts healing within 12–24 weeks (57), though there is scant evidence to support per cent area reduction in isolation as a surrogate for complete DFU healing in routine clinical practice (8).

### 4.3 Surface assessment

An analysis of surface features, textures, and segmentation may be used for understanding wound structure and valuable predictor of healing status in DFUs (58). They provide objective, quantifiable data on wound progression, including color classification (27, 30, 31), granulation area (RYB distinction) (29), texture feature (30), siamese neural network features (32), Mask R-CNN for wound segmentation (41), changes in ulcer perimeter and surface (40) and scattering analysis for detecting wound bed and surrounding tissue (33).

## 5 Characteristic wound healing prediction model

Over the past ten years, various studies have utilized prediction models with non-invasive imaging techniques. According to the list of articles that met the inclusion criteria of the current review, nine out of seventeen papers were published in clinical journals, while the remaining eight appeared in engineering journals. According to the list of articles that met the inclusion criteria, the clinical journals predominantly used statistical approaches as the foundation for prediction tasks, although some studies still relied on manual analysis by clinicians to validate healing progression. In engineering journals, most studies used machine learning strategies to predict healing progression. Table 4 shows the characteristic wound healing prediction model assessed by non-invasive imaging.

**Table 4:** Characteristics assessed by non-invasive imaging.

Non-invasive imaging	Characteristic assessed
3D imaging	Wound size (25, 26), perimeter area (25, 26), surface area (25, 26), volume (25, 26)
Photographic imaging	Color classification (27, 30, 31) Wound size (28, 29), granulation area within wound boundaries (RYB distinction) (29), texture feature (30), Siamese NN features (32)
Hyperspectral imaging	Tissue oxygenation (33–38), scattering analysis (33), oxygen saturation (34, 37)
Thermal imaging	Temperature of different wounds (36, 39), Changes in ulcer perimeter and surface (40, 41)

### 5.1 Statistical model analysis

Statistical methods were selected based on the assumption that the healing of DFUs follows a linear pattern in wound area reduction (25) (26, 54, 59, 60). Jorgensen et al. (25) compared the changes in 2D and 3D area measurements using statistical approaches. Linear regressions were used to estimate the mean changes from each patient, with the negative values as healing (decreasing in size) and positive values as nonhealing (increasing in size). The paired Wilcoxon rank test was used to test the zero median difference of the slope between 3D and 2D area measurements. The Bonett-Price approach was then used to provide the confidence interval from that median difference and the Spearman correlation coefficient was used to calculate the correlations between changes in 2D and 3D area measurements (25). Data from 150 wounds revealed that while 2D and 3D area measurements changed in the same direction, the magnitude of changes in 3D measurements was consistently greater than those observed in 2D measurements (25).

Malone et al. (26) investigated the relationship between mean wound healing measurement variables obtained from five 3D wound measurements from 21 participants using linear regression and Pearson's correlation coefficient. The performance analysis metrics were the linear healing slope and statistical significance, with a *p*-value of 0.0001 and an *R*-value greater than 0.70, respectively.

Kartika et al. (29) proposed an alternative statistical analysis approach for assessing the wound healing process in DFUs. The researchers quantified wound area, granulation area, granulation index, and respective temporal changes using ImageJ software, a free web-based software that allows easy editing, display, and analysis of images. The statistical analysis was subsequently performed to evaluate the healing progression using SPSS version 20. There were statistically significant differences in granulation index on day 3, day 7, and day 14 (*p* < 0.05).

Yang et al. (34) used HSI to capture reflectance spectra from tissue and analyzed them to predict wound healing by examining SpO2 levels. The study compared two approaches: SpO2 measurement and Principal Component Analysis (PCA). The PCA, which reduces dimensionality by identifying principal components that capture relevant features of the data, outperformed SpO2 in predicting the healing by the 12th week. While both methods showed high specificity, PCA achieved higher sensitivity (87.5% vs. 50%) and better overall predictive accuracy, with a positive predictive value of 91.3%. The study demonstrated that PCA, using HSI data, was more effective than traditional SpO2 analysis in predicting the healing of DFUs.

In 2022, Lavery et al. (35) evaluated HSI from 23 wounds to compare continuous variables and healer vs. non-healer status using the paired *t*-test and independent *t*-test, respectively. Predictive features were derived from baseline data and weekly trends, focusing on achieving a ≥50% reduction in wound area (healer), improvements in perfusion, and significant changes in cytokine levels.

Kounas et al. (38) employed the two-sample *t*-test to distinguish between groups, a binary logistic regression analysis comparing healers and non-healers based on oxy-Hb levels. There was a negative correlation between oxyHb levels at Visit 1 and the percentage of ulcer size reduction from Visit 1 to Visit 4 (*r* = −0.46, *p* = 0.02), and between oxyHb levels at Visit 2 and the percentage of ulcer size reduction from Visit 2 to Visit 4 (*r* = −0.65, *p* = 0.001).

Lopez-Moral et al. (37) applied logistic regression to predict DFU healing status using clinical and imaging-derived features. Key predictors included Transcutaneous oximetry (TcpO2), HSI parameters i.e., Skeletal Muscle Oxygen Saturation (StO2), Thermal Hyperspectral Imager (THI) parameters, Near Infrared Perfusion Index (NIR-PI), Tissue water index (TWI), and vascular indices [i.e., ankle-brachial index (ABI) and toe-brachial index (TBI)]. *P*-values below 0.05 were considered statistically significant, with a 95% confidence interval (CI). Model performance was assessed via receiver operating characteristic (ROC) curve analysis, identifying optimal cut-off values for each predictor to maximize sensitivity and specificity. TcpO2 demonstrated the highest diagnostic accuracy (AUC = 0.989, *p*-value = 0.005), followed by StO2 (93% sensitivity and 71% specificity), establishing it as the strongest independent predictor of healing.

In a thermal imaging study, Aliahmad et al. (39) employed the nonparametric Kruskal–Wallis test to investigate the relationship between the healing status of ulcers at week 4 and the ratio of DFU parameters measured in the first two weeks of ulceration (week 2 to week 1 ratio) from the isothermal areas. They defined a timely healing ulcer trajectory as showing more than a 50% reduction in area by week 4, an indirect measure routinely used.

A study conducted by Glik et al. (40) explored the impact of Hyperbaric Oxygen Therapy (HBOT) on wound healing using a combination of planimetry and thermal imaging. Statistical analysis was based on the demographics, age of the treatment group, and the median values and ranges of the collected data. To evaluate relationships between variables, Spearman's rank correlation coefficient was calculated. For normally distributed data, paired-sample *t*-tests were employed, while Mann–Whitney *U* or Wilcoxon tests were used for data that did not follow a normal distribution. Levene's test was utilized to assess data normality. When comparing more than two groups, analysis of variance (ANOVA) was applied, with a significance threshold set at *p* < 0.05.

### 5.2 Machine learning/deep Learning analysis

Recent advancements in machine learning and deep learning have significantly improved DFU monitoring and healing prediction. Various models have been developed to automate ulcer assessment, optimize treatment strategies, and enhance clinical decision-making. These approaches incorporate techniques like Random Forests, Support Vector Machines, Artificial Neural Networks, Siamese Neural Networks, and Convolutional Neural Networks, each utilizing different imaging modalities and features to provide accurate, non-invasive evaluations of wound healing.

Kim et al. (30) proposed the two machine learning models to predict the results of wound healing: Random Forests (RF) and Support Vector Machines (SVM) with RBF kernel. A total of 208 samples were used, with 25% allocated for testing and 75% used for 3-fold cross-validation training. A randomized grid search with three-fold cross-validation was used to optimize the models on the training set. Two thousand combinations of hyperparameters, such as bootstrapping, tree selection criteria, maximum depth, minimum samples per leaf, minimum samples to split a node, and number of PCA components, were tested for RF.

The PCA components, gamma (kernel coefficient), and C (error term penalty) were among the 2028 combinations tested for SVM. The optimal hyperparameters were chosen for each model from 300 randomly selected combinations to increase efficiency. The area under the receiver operating characteristic (AUROC) curve was used to assess the models' performance. To ascertain feature importance for prediction, a different RF model was trained on clinical and handcrafted image features without PCA. When trained with hand-crafted imaging features alone, both the RF and SVM models performed better than when trained with clinical or deep learning-based features alone (*P* < 0.05).

Motta et al. (31) developed an Artificial Neural Network (ANN) for classifying wound tissue into granulated, fibrin, and necrotic categories. The ulcer area was manually selected by a professional expert for use during training. A multilayer perceptron feedforward network with ten hidden neurons was employed. The Healing Ulcer Index (HUI), which quantified treatment outcomes, was designed for long-term comparison and to aid in monitoring patients at treatment centers. These indexes were based on changes in the wound area and variations in the proportions of different ulcer tissues.

Toofanee et al. (32) presented a novel framework utilizing Siamese Neural Networks (SNN) to monitor and assess the progression of DFU healing. The process began with data acquisition and preprocessing, including resizing and augmentation to address class imbalances. The framework trained an SNN using EfficientNetV2S and Vision Image Transformers to extract features and implement similarity learning, comparing new DFU images against class anchors—average feature representations for “none,” “infection,” “ischemia,” “both,” and “healthy” categories. During the evaluation, baseline images were compared with subsequent ones, generating radar charts and similarity scores to track healing progress and guide clinical decision-making. The model achieved a Macro F1-score of 0.6455, outperforming others in DFU classification tasks, and was validated by the end user, showing its utility for tracking DFU evolution.

Doulamis et al. (36) proposed a photonics-based device for DFU monitoring that integrates HSI and thermal imaging. This study has used the deep learning framework, including convolutional neural networks (CNNs), to implement the automatic segmentation and classification of pathological regions. The system supports dual configurations: a low-cost in-home version equipped with RGB and low-cost HSI sensors for patient self-monitoring, and a professional edition incorporating high-resolution HSI, near-infrared (NIR) fluorescence, and thermal sensors for clinical use. Data acquisition, processing, and interpretation were managed through an embedded software platform that integrates sensor input with machine learning models to deliver real-time, data-driven diagnostic support.

Sharma et al. (41) employed a deep learning-based approach using Mask R-CNN to evaluate DFU healing. The methodology integrated thermal and visual images through an HSV-based image fusion technique. Mask R-CNN, an instance segmentation model, accurately identifies and delineates the ulcer region, using a Region Proposal Network (RPN) and a refined ROI-Align operation. The model outputs both mask and class predictions independently, optimizing segmentation accuracy. This automated system correlates closely with clinician measurements (92.5% agreement).

The application of machine learning/deep learning in DFU imaging demonstrated the variety of algorithms for specific tasks such as classification and segmentation. Traditional machine learning models like Random Forests and Support Vector Machines used handcrafted features and improved their performance by tuning settings (e.g., randomized grid search) and reducing feature numbers (e.g., PCA). Deep learning approaches, including CNN, SNN, and Mask R-CNN learned features automatically from data, and used techniques like data augmentation to handle small datasets. The performance was measured using AUROC and F1-score, and the results were compared with expert labels as ground truth.

## 6 Limitation of DFU wound healing prediction using image techniques methods

**Figure 3:**
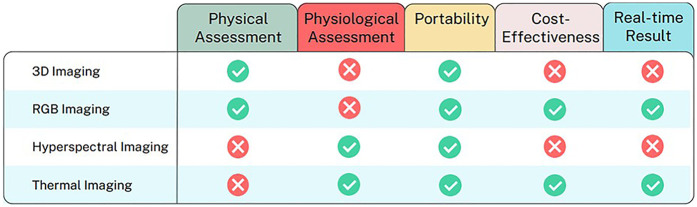
Comparative analysis of image techniques for DFU healing progression analysis across key parameters.

We have identified five key parameters to compare the technologies reviewed in this study and also the strengths and weaknesses for each non-invasive image techniques, which can be seen in Figure 3 and Table 5 respectively. The information is used as a guide to identify the strengths and limitations of each group of imaging techniques based on the articles included in this study. Based on the review, we have discovered the current limitations in the system reported over the past ten years. These limitations can be grouped into three main areas, as outlined below.

**Table 5:** The strength, weakness and technology readiness level of image technique for DFU wound healing prediction.

No	Imaging Technique	Image properties	Diagnostic value	Strengths	Weaknesses	TRL (Technology Readiness Level)
1	3D Imaging	3D WAM camera (capture 3D images)	Measure the wound area, volume, and track over time (weekly) to see how the wounds are healing.	Clearly shows the edges and shape of the wound.	Longitudinal measurement needed (≥8 weeks). Assumes linear healing, so it may misread changes if healing isn’t steady.	5–6
2	Photograph	Smartphone/tablet camera	Measure wound area, granulation area, granulation index, and nicrotic tissue. Assessing healing through the delta change of the measurement parameters.	Widely available. Can capture wound size, granulation index, and color evolution. Accessible to non-expert users like clinicians.	RGB-based monitors the surface change, lack information about depth, or metabolic insights that are important for predicting healing. RGB is highly sensitive to lighting that can destroy color and segmentation	6–7
3	Thermal imaging	FLUKE-Tir1 infrared thermos-imaging, FLIR camera	Measure the temperature of the wound bed and the area of the isothermal patch of the wound.	Can calculate the mean temperature of the wounds as an indicator of inflammation.	Need to combine with other image techniques (e.g., photograph imaging) in the wound segmentation process. Thermal reading can be affected by room temperature and recent physical activity	4–5
4	Hyperspectral imaging	HyperView camera.	Analysis of oxyhaemoglobin level.	Provide biomarker-level insights into tissue oxygenation.	Costly and less portable. Relies on consistent lighting and calibration to ensure accuracy.	7–8

TRL 1–3, basic principles observed to proof-of-concept; TRL 4, technology validated in lab; TRL-5, technology validated in controlled research environment; TRL-6, technology demonstrated in a relevant clinical environment; TRL-7, system prototype in operational environment; TRL-8, system complete and qualified.

### 6.1 Limited data for model development

The lack of accessible wound images restricts the amount of data available for training and validating predictive models. As can be seen in Table 6, the sample sizes available in most of the included papers were relatively small, often around twenty, which may not be sufficient for machine learning approaches. Therefore, it is understandable that most of the predictions or measurements of healing were processed statistically. However, large and accessible datasets would be extremely valuable for improving the accuracy of predicting wound healing status in DFUs. Researchers may struggle to create models that generalize well across different populations and clinical scenarios in the absence of a diverse and comprehensive dataset.

**Table 6:** Characteristic wound healing prediction model assessed by non-invasive imaging.

Year	Ref	Predictive characteristics	Model	Sample size	Model performance	Period of assessment	Image properties
3D (photographic) imaging							
2020	Jørgensen et al. (25)*	A negative change in the wound area	Linear regression	150 patients	Corr = 0.94	8 weeks (weeks 2, 4 and 8)	3D-WAM camera (captures 3D images), iPhone 5s for 2D images and analysed with ImageJ software.
2020	Malone et al. (26)*	Wound size reduction	Linear regression	21 participants	Healing slope = >0.7	weekly	3D wound imaging system with adhesive optical target for calibration.
Photographic imaging							
2015	Wang et al. (27)**	Wound size reduction	K-means	64 images	N/M	N/M	Nexus 4 smartphone camera.
2018	Plodere et al. (28)*	Wound size	N/M	11 participants	N/M	N/M	Samsung Galaxy S4 with OpenCV for image analysis.
2020	Kartika et al. (29)*	Delta change of granulation area, wound area, and granulation index granulation tissue,	Statistical analysis (SPSS)	30 patients	*Δ* granulation index on day-3, 7 and 14 (*p* < 0.05).	Baseline, days 3, 7 and 14.	The software used for analysis was ImageJ, which analyzes wound granulation through color segmentation and edge tracing​
2020	Kim et al. (30)**	Color and texture feature; DL features (ResNet50), clinical atribute	Random Forest (RF) and Super Vector Machine (SVM)	113 patients	AUC = 0.760–0.794	N/M	Smartphone/tablet camera,
2020	Motta *et al*. (31)**	Color of scar tissue (granulation, fibrin, and necrotic tissue)	Multilayer perceptron feedforward ANN with ten hidden layer neurons.	10 participants	Avg error compared with ImageJ = 7.48%	2 months	Sony Cybershot camera, 16.2 megapixels. Images were taken under specific lighting conditions without flash, using a reference square for scale.
2023	Toofanee et al. (32)**	Cosine similarity and euclidean distance	Siamese Neural Network (SNN)	5,734 test images	Macro F1-score = 0.6455		Images obtained from DFUC2021 challenge and additional healthy images from Kaggle.
Hyperspectral/imaging							
2015	Jeffcoate et al. (33)*	Tissue oxygenation and scattering analysis	Regression	43 people	The positive correlation between oxygenation assessed by HIS and time to healing (*P* = 0.03)	12 and 24 weeks	A custom-built hyperspectral camera was used to measure light reflection and calculate oxygen levels in the skin.
2018	Yang et al. (34)**	Oxygen saturation (SpO2) levels in tissue and PCA scores	PCA	43 participants	PCA: Sensitivity = 87.5%, Specificity = 88.2%) SpO2: Sensitivity = 50%, Specificity = 88.2%	12 weeks	A Peltier-cooled charge-coupled device (CCD) Sensicam QE coupled with an ImSpector V10E imaging spectrograph.
2020	Lavery et al. (35)*	Impact of continuous diffusion of oxygen (CDO) therapy	Statistical analysis (paired *T*-tests and independent *T*-tests)	23 patients	N/M	3 weeks	Camera type: Hyperspectral imaging device (HyperView, Hypermed) and transcutaneous oxygen measurement (PeriFlux 5000). Measurement device: 3D wound measurement using inSight (eKare).
2021	Doulamis *et al*. (36)**	Tissue oxygen saturation (SpO2, StO2), peripheral blood flow and arterial perfusion, temperature differences	CNN and decision trees	N/M	N/M	19 months	Hyperspectral Sensor: IMEC-based sensors using Fabry–Pérot structures for capturing wavelengths between 460 and 975 nm.
2022	López-Moral et al. (37)*	Oxygen saturation (StO2)	Logistic regression	21 patients	StO2: Sensitivity = 93% Specificity = 71% AUC = 0.932	24 weeks	High-quality infrared-enhanced CMOS megapixel camera sensor (CMOSIS CMV 2000 3E12) integrated into an intelligent camera with USB3-data transfer
2023	Kounas et al. (38)*	Oxyhemoglobin (OxyHb), Deoxyhemoglobin (DeoxyHb), and Oxygen Saturation (O2Sat) levels.	Statistical analysis: Logistic regression	27 patients	Visit 1: Sensitivity = 85%, Specificity = 70% Visit 2: Sensitivity = 85% Specificity = 85%	12 weeks, visits every 3–4 weeks	HyperView® apparatus.
Thermal imaging							
2019	Aliahmad et al. (39)**	Temperature of wound bed area, area of the isothermal patch of the wound bed, area of the isothermal patch of the peri-wound, number of isolated isothermal patches of the wound region, and physical wound bed area from color images	Statistical analysis: nonparametric Kruskal–Wallis tests	26 patients	The ratio of wound bed at week 2 with the based line: (*P* = 0.036)	12 weeks	Fluke-TiR1 infrared thermo-imaging and Nikon D90 DSLR cameras,
2019	Glik *et al*. (40)*	Effects of hyperbaric oxygen therapy (HBOT) on ulcer healing	Statistical analysis (ANOVA, paired-sample *t*-test)	142 patients	N/M	N/M	Thermovision Camera E60 (FLIR Systems, Sweden), calibrated using black body standards for thermal imaging​
2023	Sharma et al. (41)**	Ulcer-to-Foot Area Ratio (U: F Ratio), Absolute Temperature Difference (ATD), Mean Temperature Distribution	Mask R-CNN	42 patients	Accuracy = 92.50%	12 weeks	A FLIR E-60 infrared thermal imaging camera (which gives thermal as well as RGB images)

* Clinical article.

** Engineering article.

### 6.2 Automated wound segmentation methods

In the clinical environment, efficient computational methods are needed to obtain quick and real-time results, including automated wound segmentation. Manual segmentation makes it unsuitable for the device to be used in the clinical setting, delays the process of analysis, and leads to data without clear standards, as different individuals might produce varying tracing results (25, 30).

### 6.3 Technical limitations of imaging devices

Technical limitations must be considered before the device can be used in a wide range of clinical settings. Some studies have used complicated tools for data collection that are not portable or feasible for routine use in primary care settings (25, 27, 34, 41). Expensive imaging technologies such as HIS (25, 37) can also prevent many healthcare providers from using advanced tools to assess DFUs, especially since these devices are intended for use not only in clinical environments but also in home care settings.

## 7 Future directions and open research issues

The future direction of wound diagnostics should focus on supporting healthcare providers while leading towards enabling individuals with wounds to self-monitor. Achieving this would requires a coordinated roadmap that addresses key research gaps across data infrastructure, clinical validation, technology development, and clinical integration. With the growing potential of machine learning and non-invasive imaging, the following roadmap outlines the critical steps to advance predictive wound healing for DFU using non-invasive imaging techniques.

### 7.1 Build large-scale, multicenter datasets

Currently, there is a lack of large-scale datasets for DFU images. This represents a bottleneck in advancing predictive models for healing. Future efforts should focus on developing centralized, open-access repositories of annotated wound images that are collected from diverse populations and clinical settings (61–63). These repositories would enable researchers to train and test predictive models on a broader set of data, significantly improving the generalizability and robustness (64).

Techniques like synthetic data generation using methods such as generative adversarial networks (GANs), could also be explored to augment existing datasets and overcome data bias. Synthetic wound images have shown promise in enhancing dataset diversity, particularly in addressing class imbalances and rare scenarios (24, 65).

Combining wound imaging with complementary information such as patient demographics, comorbidities, biochemical markers, and treatment history could provide a more holistic understanding of the healing process. Multimodal approaches have been shown to improve model performance in predicting the healing of DFU (66) and chronic wounds (67). This integration would require collaborative efforts across disciplines to standardize data collection protocols and ensure interoperability between datasets.

### 7.2 Standardize imaging protocol

Standard protocol in non-invasive image technique to predict healing status of DFU is essential to ensure consistent, reliable, and reproducible assessment. Consistent procedures for image acquisition such as lighting, positioning, calibration, environmental/camera control are critical to ensure reliable data across time and clinical settings. Protocol standardization can facilitate clinical implementation by making imaging more interpretable for healthcare providers.

### 7.3 Integrate machine learning for image analysis

Machine learning methods, especially convolutional neural networks (CNNs), such as U-Net and its variants, have proven highly effective in medical image segmentation and could be adapted for use in predicting DFU healing (68, 69). To enhance clinical adoption, these methods must be computationally efficient, capable of running on standard hardware, and robust to variations in wound appearance caused by lighting, skin tone, and ulcer severity.

In addition, the implementation of explainable AI (XAI) methods in segmentation tools would enhance the transparency and acceptance among clinicians (70). Providing visual overlays or confidence maps could help clinicians validate the automated segmentation results, ensuring the reliability in decision-making processes.

### 7.4 Conduct multicenter clinical trials

To validate the predictive utility of imaging and AI models, multicenter prospective clinical trials are needed. These should evaluate whether the early prediction of healing status leads to improved patient outcomes, such as earlier intervention or reduced amputation. Trials should also assess how imaging tools perform across different care settings, such as hospitals, community, and home care services.

### 7.5 Develop portable and user-friendly imaging devices

Future research should focus on developing portable, cost-effective devices that maintain high diagnostic accuracy. Advances in sensor miniaturization, wearable technology, and smartphone-based imaging platforms could provide practical solutions, enabling real-time wound assessment and monitoring in diverse care environments (71, 72). In addition, the integration of user-centered design principles is critical to ensure that these tools are intuitive for non-specialist users, such as general practitioners and caregivers (73, 74). Efforts should also be made to reduce the computational requirements of data analysis algorithms, enabling deployment on low-power devices.

### 7.6 Enable multimodal imaging integration

Combining multiple image techniques into an integrated framework provides a more comprehensive view of wound healing status by capturing physical, physiological, and biochemical information. These can improve diagnostic capabilities beyond the limitations of individual techniques. Future direction should focus on developing tools that can quickly and accurately analyze the combined imaging features, which make it easier for clinicians to assess wounds and make treatment decisions.

## 8 Discussion and conclusion

Assessing wound healing progression is crucial in DFU to ensure treatment effectiveness, detect complications early, and guide timely clinical decisions. It helps prevent serious outcomes like infection or amputation and improves patient recovery and care quality. In clinical practice, most clinicians still rely on visual assessment to monitor DFU healing due to simplicity and cost-effectiveness. In recent studies, non-invasive imaging techniques offer safer, affordable, and portable alternatives to conventional methods like MRI, CT, and PET in the healing prediction of DFU. They enable real-time monitoring and early prediction using Machine learning/deep learning analysis, however, they are not yet widely adopted in routine care because of the validity and accessibility.

This systematic review evaluated the current non-invasive imaging techniques used to predict the wound healing status of DFUs. Our results highlight the wound healing classification process, though it remains in the early phases of development and clinical validation. We identified several imaging methods, such as visible light imaging, 3D imaging, hyperspectral imaging, and thermal imaging. Each technique presents its own strengths and faces different problems in development and application. In order to provide a tool that can be used in clinical, home, and community services, several criteria must be met, including a clear data collection protocol; a large number of data samples; a process for extracting information from the wound area, and for classifying the feature information into accurate outputs of healed and non-healed wounds; and the possibility of being developed in real-time analysis in a short time.

While non-invasive imaging techniques hold significant promise for predicting diabetic foot ulcer (DFU) healing, several barriers burden their routine use in clinical practice. Overcoming these obstacles is crucial for successfully translating these promising technologies into everyday patient care.

1. **Regulatory barriers:** Regulatory approval for new medical technologies presents a significant challenge. The process of approving medical devices and technologies can be time-consuming and costly. Imaging technologies require extensive validation and clinical trials before they can be used in practice. The different standards in clinical validation require multiple rounds of testing that can delay the introduction of promising technologies to be used. Aligning regulations might help speed up approval and reduce the costs.
2. **Technological barriers:** Challenges such as the lack of standardized image protocols, and the complexity of some technologies (e.g., hyperspectral and 3D imaging) make these tools difficult to integrate into clinical workflows. Additionally, some of these technologies require specialized equipment and expertise that are not universally available. Developing a standardized imaging protocol and improving compatibility with existing healthcare systems may make integration easier.
3. **Economic barriers:** The high cost of advanced imaging equipment and computational resources needed for data analysis is a significant barrier. Non-invasive imaging technologies can be expensive and may limit their accessibility for use in home or community care. Conducting cost-benefit analyses and improving affordability might help increase accessibility.
4. **Clinical barriers:** Clinicians may face difficulties in adopting these new technologies due to a lack of training or resistance to change. There is a need for robust clinical validation to demonstrate the reliability and clinical utility of these imaging techniques before they can be widely accepted. Providing comprehensive training programs and demonstrating the clinical benefits might encourage adoption.

This review identifies which imaging techniques are most suitable for routine use in clinical, home care, and community-based care settings for predicting DFU healing outcomes. As the findings were based on only 17 eligible studies, this focused selection allowed for detailed analysis of current imaging techniques, providing valuable insight and highlighting important directions for future research.

Based on our findings, we conclude the following:

1. **Need for end-to-end integration:** To achieve real-time results, it is necessary to build an end-to-end integrated system, from image acquisition to analysis and output. From this perspective, visual (RGB) imaging and thermal imaging are promising alternatives due to the simplicity and the ability to process data automatically on the same device without human assistance (e.g., through automatic segmentation and classification). These methods are also more portable and user-friendly compared to more complex technologies like hyperspectral imaging or 3D imaging, making them better suited for use in home and community-based settings.
2. **Role of AI in early prediction**: With the integration of advanced techniques such as machine learning and deep learning, early prediction of healing status is possible (no need to wait up to 4 weeks to ascertain wound healing ability). This can assist clinicians in making more timely and accurate treatment decisions.
3. **Need for validation and clinical readiness**: While non-invasive imaging techniques have high potential as alternatives to monitor wound healing progress, further validation supported with larger datasets is still needed to ensure the reliability of the results before these techniques can be translated into clinical practice. Additionally, more detailed consideration of the barriers to clinical adoption, such as clinician training, and integration into existing clinical workflow, would be beneficial to fully understand what is required to implement these technologies in routine care.

## Data Availability

The original contributions presented in the study are included in the article/Supplementary Material, further inquiries can be directed to the corresponding author.
